# Commentary on: Bai, X., Zhao, N., Koupourtidou, C., Fang, L.-P., Schwarz, V., Caudal, L.C., Zhao, R., Hirrlinger, J., Walz, W., Bian, S., Huang, W., Ninkovic, J., Kirchhoff, F., Scheller, A. “In the mouse cortex, oligodendrocytes regain a plastic capacity, transforming into astrocytes after acute injury”

**DOI:** 10.1007/s00424-023-02846-4

**Published:** 2023-07-31

**Authors:** Friederike Pfeiffer

**Affiliations:** Department of Neurophysiology, Institute of Physiology, Eberhard Karls University of Tübingen, Tübingen, Germany

In the central nervous system (CNS), cells of the oligodendroglial lineage represent a source of regenerative potential, the extent of which currently is not entirely understood. The regenerative potential of the oligodendrocyte precursor cells (OPCs, or NG2 cells), which reside in the CNS throughout the entire lifespan of vertebrates, keeping their ability to proliferate in order to increase their population, or to differentiate and give rise to myelinating oligodendrocytes, has been extensively studied and described [3]. However, much less is known about the plasticity of mature oligodendrocytes, and whether conversion between different macroglial cell types can occur.

During embryonic development, cells of the astrocytic and oligodendrocytic lineage evolve from a common progenitor [14]. Although they highly interact with each other to support development and function of the CNS [8], astrocytes and oligodendroglial cells are regarded as distinct macroglial populations with their own set of specific functions in the postnatal brain. Some overlap between the cells of the astrocytic and oligodendroglial lineage has already been recognized: (i) in the late embryonic ventral forebrain, a subset of OPCs was shown to generate a subpopulation of protoplasmic astrocytes [17]; (ii) whereas OPCs generate oligodendrocytes in the healthy postnatal brain, reactive astrocytes can arise from OPCs upon injury, e.g., in the adult spinal cord [1, 9]. However, the extent of this lineage conversion is highly variable depending on the model and injury paradigm used, and the majority of cells arising from OPCs in injury models are still the cells of the oligodendroglial lineage [4, 12].

In their recent paper published in *Developmental Cell*, Bai et al. describe a thus far unknown plastic cell type arising from mature oligodendrocytes after CNS injury, which they call “AO cells” [2]. They initially identified these cells in several mouse lines expressing fluorescent reporter molecules for either oligodendrocytes or astrocytes after cortical stab wound injury, but not in control uninjured brains. Using the split-cre system, which has been invented by the Kirchhoff lab several years ago [7], simultaneous activation of oligodendrocytic PLP and astrocytic GFAP promoters in the AO cells upon stab wound injury could be demonstrated. The majority of these oligodendrocyte-derived AO cells subsequently became astrocytes (the remainder of the cells became oligodendrocytes or OPCs), reminiscent of O-2A progenitor cells that have been described in the 1980s under in vitro conditions [13] and in cultured optic nerve cells [5].

By single-cell RNA sequencing of the injured cortical tissue, the authors confirmed the existence of a subpopulation of mature *o*ligodendrocytes that activated *a*stroglial genes as a response to stab wound injury—hence leading to the term “AO cells” [2]. Using mice simultaneously expressing fluorescent markers for oligodendrocytes and astrocytes (e.g., PLP-DsRed1/GFAP-EGFP mice), the authors were able to follow cells during their conversion from an oligodendrocyte into an astrocyte by in vivo 2-photon microscopy for up to 30 days. Furthermore, these cells incorporated the proliferation marker BrdU, connected to adjacent cells via gap junctions and contacted blood vessels with their endfeet, thus exerting the functions of astrocytes. Additionally, Bai et al. also confirmed the presence of AO cells in two stroke models.

Finally, IL-6 released by pro-inflammatory microglia was found to promote the formation of AO cells from oligodendrocytes. Besides their important role in CNS injury, microglia have more recently been shown to regulate myelin formation [11], as well as an increase in the number of OPCs after demyelination [16], and may therefore play a key role in the generation of AO cells.

This new demonstration of inter-lineage plasticity is highly interesting, as it adds a novel player to the brains’ already described resources for plasticity and regeneration. While in rodents it is generally assumed that the proliferation of OPCs and their subsequent differentiation into oligodendrocytes is responsible for remyelination after injury, analysis of human tissue revealed that in humans remyelination may be carried out by old, already existing oligodendrocytes, and it is not yet clear which mode of remyelination predominates in human brains [6]. Of note, the plasticity of oligodendroglial cells is often studied in demyelinating diseases or models, whereas Bai et al. used stab wound injury and stroke models, thus looking at a different cause of cellular reorganization. Plasticity in the sense of dedifferentiation of mature myelinating cells has thus far only been described in lower vertebrates [10] or in the peripheral nervous system, where Schwann cells are able to dedifferentiate in response to axonal injury [15]. The conversion of mature oligodendrocytes into astrocytes represents a new type of plasticity.

In the future, a more detailed characterization of this interesting phenomenon will shed light on the nature and potential of these AO cells, the signals by which they are induced and/or regulated, the circumstances and the frequency with which they occur as well as their contribution to brain repair. We will be looking forward to learn more about the regenerative potential of oligodendrocytes in different injury or aging conditions. There may be even more inter-cellular plasticity as has been anticipated to date, with hopefully more targets to be discovered for the development of novel therapeutic approaches.

## Data Availability

Not applicable
